# Editorial: Plant-fungal interactions

**DOI:** 10.3389/fmicb.2023.1236394

**Published:** 2023-07-25

**Authors:** Dong-Qin Dai, Nakarin Suwannarach, Thushara Chathuranga Bamunuarachchige, Samantha Chandranath Karunarathna

**Affiliations:** ^1^Center for Yunnan Plateau Biological Resources Protection and Utilization, College of Biological Resource and Food Engineering, Qujing Normal University, Qujing, China; ^2^Center of Excellence in Microbial Diversity and Sustainable Utilization, Chiang Mai University, Chiang Mai, Thailand; ^3^Department of Biology, Faculty of Science, Chiang Mai University, Chiang Mai, Thailand; ^4^Department of Bioprocess Technology, Faculty of Technology, Rajarata University of Sri Lanka, Anuradhapura, Sri Lanka; ^5^National Institute of Fundamental Studies (NIFS), Kandy, Sri Lanka

**Keywords:** agriculture, biotechnology, biopesticides, endophytes, biofertilizers

Fungi are known to be older than plants, while plant-fungal interactions are as old as the evolutionary period of higher plants (Zeilinger et al., 2016). Some fungi interact positively or negatively with plant roots in the rhizosphere or aboveground parts of the plant (Barea et al., 2005). These fungi have different lifestyles viz., saprophytic, pathogenic, endophytic, or symbiotic, but the differences among different lifestyles are not always apparent (Błaszczyk et al., 2021). The beneficial plant-associated fungi assist their hosts by stimulating their growth, producing secondary metabolites, and improving their resistance to biotic and abiotic stresses (Verma et al., 2022). In contrast, pathogenic fungi cause diseases and are one of the major threats to crop yield and food security (Xu, 2022).

The fungi-plant interactions ranging from harmful to valuable associations, play a leading role in natural and agricultural ecosystems (Balestrini, 2021). These interactions affect agriculture, the environment, and ultimately the economy. Many fungi are yet to be discovered; thus, studying plant-fungal interactions is very important for finding these missing fungi and helpful for exploring their roles in the ecosystem (Antonelli et al., 2020). Studies should be conducted predominantly in the tropics since these regions have a high plant diversity that positively correlates with fungal diversity (Shen et al., 2021). Nevertheless, previous studies showed that even temperate plant species harbor many cryptic species. Hence, exploring the inhabiting fungi from the perspective of plant-fungal interactions in temperate and tropical regions is essential.

To create and spread a better understanding of this exciting area of research, we proposed the Research Topic “*Plant-fungal interactions*.” In this Research Topic, we accepted 25 articles, including 23 original articles and two reviews on different aspects of plant-fungi interactions. An overview of the scientific content is summarized below.

Lu et al. presented original research on how arbuscular mycorrhizae influence raspberry growth and soil fertility under conventional and organic fertilization. The results showed that coupling organic fertilizers and bioinocula, including diverse arbuscular mycorrhizae species, enhance raspberry growth and soil fertility. Thioredoxin VdTrx1 (an unconventionally secreted protein with biological functions inside and outside of the cells) in fungal tissues involves scavenging intracellular reactive oxygen species and sulfite assimilation. Tian et al. showed that Thioredoxin VdTrx1 is a virulence factor in *Verticillium dahlia*. The results further confirmed that VdTrx1 is necessary for the full virulence of *V. dahliae* on susceptible hosts. Mangrove-associated microorganisms have received increasing attention due to their important ecological roles and the wide range of services they provide to the environment and economy. Zhu et al. used high throughput sequencing of the internal transcribed spacer 2 (ITS2) to assess epiphytic and endophytic phyllosphere fungal communities of six true mangrove species and five mangrove associates. The results of this study highlight the important role of plant phylogeny in the assembly of epiphytic but not endophytic fungal communities in mangrove ecosystems. To understand the resistance mechanisms of the *Ganoderma lingzhi* response to *Trichoderma hengshanicum* infection, Wang T. et al. observed *G. lingzhi* transcript accumulation at 0, 12, and 24 h after *T. hengshanicum* inoculation. The results revealed that the down-regulation of differentially expressed genes led to the inhibition of heat shock protein (HSP) function, which compromises the HSP-mediated defense signaling transduction pathway, leading to the susceptibility of *G. lingzhi*. Miang, a traditional fermented food product produced from the leaves of *Camellia sinensis* var. *assamica* is found in the hill areas of Northern Thailand. Using the culture-dependent method, Kanpiengjai et al. investigated the yeast ecology of *C. sinensis* var. *assamica* tea flowers collected from six provinces in upper Northern Thailand. They characterized the tannin tolerance ability of the isolated yeasts. This study suggests that floral nectar supports the formation of yeast communities beneficial for Miang production. Paridis Rhizoma is a Chinese medicinal herb with strong anti-inflammatory and anti-tumor properties. Chen Y. et al. showed that the inhibitory effects of fermented Paridis Rhizoma extract (PRE) on liver cancer cells (Hepal-6), cervical cancer cells (Hela), and lung cancer cells (A549) are stronger than that of the unfermented extract. *Venturia carpophila*, the causal agent of peach (*Prunus persica*) scab disease, mume (*Prunus mume*), and apricot (*Prunus armeniaca*), is widely distributed around the world. Zhou et al. carried out the genetic variation and population structure in *V. carpophila* isolated from peach, mume, and apricot in China. They found that the genetic identity of *V. carpophila* isolates depends on the host, not the geographic region. Morphological characteristics and multigene phylogenetic analyses are used as the latest techniques for fungal species identification. Tennakoon et al. introduced *Montagnula acaciae, Paraconiothyrium zingiberacearum*, and *Paraphaeosphaeria brachiariae*, as distinct new species from dead plant litter based on morphological differences and DNA sequence data. In addition, *Montagnula jonesii, Paraconiothyrium fuckelii, Spegazzinia deightonii*, and *S. tessarthra* were reported as new host records from *Ficus benjamina, Dimocarpus longan, Hedychium coronarium*, and *Acacia auriculiformis* respectively, for the first time. Furthermore, *Paraconiothyrium archidendri* and *P. brasiliense* were reported for the first time from *Magnolia* sp. in China, and *Paraconiothyrium rosae* was synonymized under *Pa. fuckelii*. Introducing exotic or non-native trees fails due to a lack of suitable fungal partners. Wang R. et al. showed that exotic *P. radiata* is a suitable tree capable of successfully getting established by interaction with the co-introduced *L. deliciosus* or local ectomycorrhizal fungi. However, care should be taken when large-scale planting of *P. radiata* is done. Plants of the genus *Iris* are widely cultivated because of their medicinal, ornamental, and economic values. However, it often suffers from *Alternaria* leaf spot or blight disease, leading to considerable losses for its commercial value. Gou et al. isolated 122 *Alternaria* strains in section *Alternaria* from diseased leaves of *Iris* spp. in 14 provinces or municipalities of China from 2014 to 2022. They introduced two novel and two known species that can induce leaf spots in *Iris*. In Sichuan province, *Juglans regia, J. sigillata*, and the hybrid *J. regia* × *J. sigillata* are the commercially important walnut plants, while *J. regia* is the most widespread walnut plant. In order to update fungi associated with Sichuan walnuts, Xu et al. surveyed 15 representative regions in Sichuan. The survey revealed 10 fungi belonging to Dothideomycetes and Sordariomycetes that were described based on morpho-molecular analyses. *Rhododendron*, an essential ornamental plant, is abundant in Yunnan Province of China, and 61 species of *Rhododendron* have been reported from Cangshan Mountain in Yunnan. Gu et al. introduced six new fungal species isolated from fresh leaves of *Rhododendron cyanocarpum, R. decorum*, and *R. delavayi* at Cangshan Mountain for the first time. *Ganoderma* is a globally distributed genus covering ecological, medicinal, economic, and cultural species. He et al. used morphology and multigene phylogeny to identify 21 specimens of *Ganoderma* collected in the Yunnan Province of China, representing 18 species. In addition, a checklist and a key to *Ganoderma* in Yunnan Province were given in the paper. Drought stress is one of the major abiotic factors that limit plant growth and cause ecological degradation. To investigate the role of arbuscular mycorrhizal fungi (AMF) on reactive oxygen species (ROS) generation and ROS scavenging ability under drought stress in *Bombax ceiba*, Li et al. carried out an experiment. The results revealed that AMF inoculation could maintain ROS homeostasis by mitigating drought-induced ROS burst via decreasing ROS generation and enhancing the ROS scavenging ability of *B. ceiba* seedlings.

Plant diseases caused by oomycetes inflict severe damage to various crops; however, biocontrol of oomycete-related diseases is poorly done. In this regard, Liu et al. used 86 *Trichoderma* isolates against *Phytophthora nicotianae, Ph. capsici, Pythium vexans, Py. ultimum*, and *Py. dissotocum* through dual culture assay and the results showed that the fungal strains exhibit strong antagonistic effects against oomycete pathogens, and those fungal strains can be integrated into disease management strategies. Multiple interactions happen between host plants and phyllosphere microbes, such as bacteria, oomycetes, and fungi. Wang K. et al. explored the interaction of *Protomyces arabidopsidicola* (isolated from phyllosphere), with Arabidopsis plant and found that *Pr. arabidopsidicola* strain C29 is pathogenic on Arabidopsis plant but can survive in its phyllosphere both in a controlled environment and under natural field conditions. Wood-associated fungi play a vital role in the degradation of wood and the recycling organic matter in forests. Luo and Zhao introduced a new fungal order Xenasmatales from Yunnan, China, based on morphology and multigene phylogeny to accommodate the family Xenasmataceae. In addition, *Xenasmatella nigroidea* and a key to *Xenasmatella* worldwide were provided. *Colletotrichum*, a widespread fungal pathogen, causes various plant diseases and *Colletotrichum fructicola* causes oil-tea (*Camellia oleifera*) anthracnose. Chen, Chen, Tan, He et al. selected eight candidate reference genes (*CfCk, CfRpp, CfUce, CfRrp, CfAdrh, CfDd, CfAct*, and *CfTub*) from *Co. fructicola. Camellia oleifera* transcriptome data and evaluated and sequenced using geNorm, NormFinder, and BestKeeper algorithms. The results revealed that CfRrp has better stability in *Co. fructicola*, during the growth and invasion of different oil-tea leaves. Wheat (*Triticum aestivum*), an important cereal crop, is widely grown in temperate zones and higher elevations. Fusarium-head blight (FHB) is a critical wheat disease throughout the world. Tang et al. identified *Fusarium avenaceum* as the causative agent of FHB disease in Linzhi City, southeast of Tibet, China, based on morphology, multigene phylogeny, and pathogenicity test. As a result of an ongoing survey of microfungi associated with *Magnolia grandiflora* in Qijing, Yunnan, China, Wijayawardene et al. introduced three new species and five new records of saprobic fungi. *Camellia oleifera* (oil tea), mainly used for producing high-quality edible oil, is an important cash crop in China. Anthracnose of oil tea is a significant disease that limits the tea oil yield. Considering this fungal disease, Chen, Chen, Tan, Mo et al. reviewed the status of the harm, pathogen types, control measures, and pathogenic molecular mechanism of oil tea anthracnose, and this review provides essential information to control oil tea anthracnose. Ectomycorrhizal (ECM) fungi play a vital role in forest ecosystems. However, little is known about the bacterial and fungal communities associated with ECM roots. Zeng et al. surveyed the bacterial and fungal microbiome of ECM roots from stone oaks (*Lithocarpus* spp.) and Yunnan pines (*Pinus yunnanensis*) in the subtropical forests of the Ailao Mountains, Yunnan, China, and the results revealed that Rhizobiales and Acidobacteriales dominate bacterial community. In contrast, the fungal community is mainly composed of Russulales and Thelephorales. Microbial co-occurrence network analysis is commonly used for data exploration in plant microbiome research. To overcome oversimplified interpretation of the networks stemming from the stereotypical dichotomy between bacteria and fungi, Lee et al. recommend; understanding the dynamics and mechanisms of co-occurrence networks through generalized Lotka-Volterra and consumer-resource models, finding alternative ecological explanations for individual negative and positive fungal-bacterial edges, and connecting cross-kingdom networks to abiotic and biotic (host) environments. Fungi secrete various effectors to control host defense systems. Yang et al. identified necrosis- and ethylene-inducing protein 1 (Nep1)-like protein (NLP) effector gene, *CgNLP1*, which contributes to conidial germination, appressorium formation, invasive growth, and virulence of *Co. gloeosporioides* in rubber tree. A fairy ring is fungal fruiting bodies that occur as a ring on a spot. Wang Q. et al. showed the presence of abundant beneficial microbes driving the flourishing growth of plants in the fairy ring soil and provided bio-resources for agricultural growth-promoting agents.

## Author contributions

SK drafted the editorial. All authors contributed to editorial revision and approved the final paper.
